# In Silico and In Vivo Pharmacokinetic Evaluation of 84-B10, a Novel Drug Candidate against Acute Kidney Injury and Chronic Kidney Disease

**DOI:** 10.3390/molecules29010159

**Published:** 2023-12-27

**Authors:** Man Su, Xianru Liu, Yuru Zhao, Yatong Zhu, Mengqiu Wu, Kun Liu, Gangqiang Yang, Wanhui Liu, Lin Wang

**Affiliations:** 1 Key Laboratory of Molecular Pharmacology and Drug Evaluation, Ministry of Education, Collaborative Innovation Center of Advanced Drug Delivery System and Biotech Drugs in Universities of Shandong, School of Pharmacy, Yantai University, Yantai 264005, China; suman999@163.com (M.S.); liuxianru1014@163.com (X.L.); y281520@126.com (Y.Z.); nkymyatong@163.com (Y.Z.); liukun@ytu.edu.cn (K.L.); oceanygq@ytu.edu.cn (G.Y.); 2 Nanjing Key Laboratory of Pediatrics, Children’s Hospital of Nanjing Medical University, Nanjing 210008, China; mengqiuwu@126.com

**Keywords:** chronic kidney disease, 84-B10, pharmacokinetic, tissue distribution, physiologically based pharmacokinetic model

## Abstract

Acute kidney injury (AKI) and chronic kidney disease (CKD) have become public health problems due to high morbidity and mortality. Currently, drugs recommended for patients with AKI or CKD are extremely limited, and candidates based on a new mechanism need to be explored. 84-B10 is a novel 3-phenylglutaric acid derivative that can activate the mitochondrial protease, Lon protease 1 (LONP1), and may protect against cisplatin-induced AKI and unilateral ureteral obstruction- or 5/6 nephrectomy [5/6Nx]-induced CKD model. Preclinical studies have shown that 84-B10 has a good therapeutic effect, low toxicity, and is a good prospect for further development. In the present study, the UHPLC-MS/MS method was first validated then applied to the pharmacokinetic study and tissue distribution of 84-B10 in rats. Physicochemical properties of 84-B10 were then acquired in silico. Based on these physicochemical and integral physiological parameters, a physiological based pharmacokinetic (PBPK) model was developed using the PK-Sim platform. The fitting accuracy was estimated with the obtained experimental data. Subsequently, the validated model was employed to predict the pharmacokinetic profiles in healthy and chronic kidney injury patients to evaluate potential clinical outcomes. C_max_ in CKD patients was about 3250 ng/mL after a single dose of 84-B10 (0.41 mg/kg), and C_max,ss_ was 1360 ng/mL after multiple doses. This study may serve in clinical dosage setting in the future.

## 1. Introduction

The kidneys are important tissues for maintaining fluid, electrolyte, and acid–base balance, and regulating and maintaining key biological mechanisms in an organism [1]. When diseases or other conditions impair kidney function, acute kidney injury (AKI) or chronic kidney disease (CKD) occurs. AKI is a sudden loss of renal function caused by various etiologies, which is manifested by the decline of glomerular filtration rate, creatinine retention, electrolyte disorder and acid–base imbalance [2]. CKD is a group of syndromes defined as chronic structural and functional renal dysfunction that persists for more than three months due to various reasons [3]. In developed countries, CKD has attracted much attention because of its high patient morbidity and mortality [4]. It is estimated that in 2017, there were 697.5 million patients with all-stage CKD globally, with a prevalence of nearly 10%. In addition, about 1.2 million patients died from CKD worldwide in 2017, and the number is estimated to increase to 2.2 to 4.0 million by 2040 [5]. CKD may progress to end-stage kidney disease and cardiovascular disease without adequate management [6]. Currently, treatment includes reducing cardiovascular risk, treating albuminuria, and avoiding potential nephrotoxins [7]. Angiotensin-converting enzyme inhibitors and angiotensin receptor blockers have been the only recommended drugs for CKD patients, but neither can reduce all-cause mortality [8]. In the past, AKI and CKD have been regarded as separate entities. Currently, more and more investigations indicate that AKI is an independent and significant risk factor for CKD development [9]. Due to similarity in some pathological processes between AKI and CKD, it is believed that AKI can lead to the progression of CKD and an increased long-term risk of end-stage renal disease (ESRD) [10,11]. It is of vital importance to discover new drugs for the treatment of AKI and CKD.

Mitochondrial dysfunction in renal cells plays a crucial role in the pathophysiology of AKI and CKD. Therefore, mitochondria have been recognized as a promising target to improve the treatment of patients with kidney diseases [12]. Currently, many mitochondrial-targeted compounds are being developed for the therapy of AKI and CKD. 5-[[2-(4-methoxyphenoxy)-5-(trifluoromethyl) phenyl] amino]-5-oxo-3-phenylpentanoic acid (CAS No.: 698346-43-9) (designated 84-B10) is a novel 3-phenylglutaric acid derivative, which has been proven to alleviate cisplatin-induced acute kidney injury through antagonizing mitochondrial oxidative stress-mediated ferroptosis [13]. Furthermore, it has been found to activate Lon protease 1 (LonP1), a major mitochondrial protease, and has exhibited remarkable therapeutic effects on mice with CKD [14]. The preclinical toxicology results suggested a wide clinical therapeutic window and that it was a good prospect for further development. Nonetheless, there is limited research on its pharmacokinetics. To date, only plasma and tissue concentrations in mice after a single intraperitoneal injection of 84-B10 (5 mg/kg) can be obtained from the literature. Undesirable pharmacokinetics is one of the main reasons for the failure of drug development, therefore, essential evaluation needs to be undertaken during the discovery stage. Currently, ADME properties can be obtained through in silico methods [15,16], but the determination of drug exposure in plasma and target tissue remains challenging.

The concept of the physiologically based pharmacokinetic (PBPK) model was proposed by Theorell in 1937, which comprises a physiological model and a drug model [17,18]. The physiological model comprises different organs of the body, linked by the circulating blood system, and is independent of the compound. The drug model consists of physicochemical parameters and biochemical properties of the particular compound [19,20]. The PBPK model allows a priori simulation of the drug pharmacokinetic profile in plasma or tissues through in silico methods, and has become an integral tool in the drug discovery stage without sufficient pharmacokinetic data from animals [21,22,23]. A validated and reliable PBPK model can assist in drug design and dosage adjustments in special populations [24]. As the tissue concentrations of the drug in humans cannot be acquired experimentally but can be predicted by the PBPK model, potential action or xenobiotic exposure can be alerted [25].

In this paper, an essential pharmacokinetic and tissue distribution of 84-B10 was studied in rats by UHPLC-MS/MS. The physicochemical and PK properties of 84-B10 were acquired via in silico methods. A “bottom-top” whole-body PBPK model was developed and the accuracy of the model was estimated using experimental data from rats and mice. The validated model was then applied to predict the pharmacokinetic profiles in healthy and chronic kidney injury patients. The paper aims to predict therapeutic benefits and adverse effects of 84-B10 through a PBPK model, following the 3R principle (replacement, reduction, and refinement) to ensure animal welfare [26].

## 2. Results

### 2.1. UHPLC-MS/MS Method Validation for the Quantification of 84-B10

**Figure 1:**
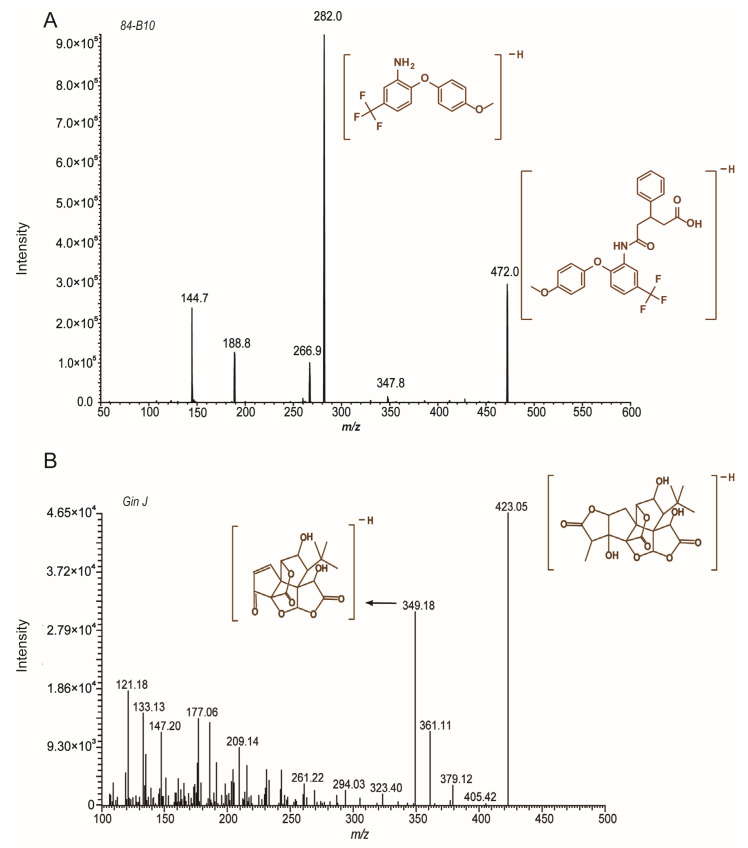
MS/MS spectra of (**A**) 84-B10, and (**B**) Ginkgolide J (internal standard) in negative mode.

**Figure 2:**
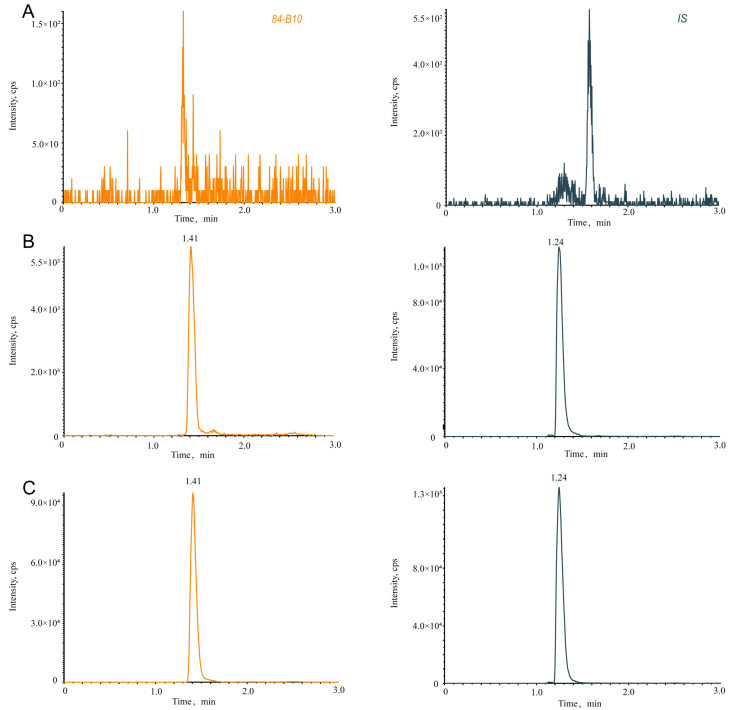
Representative MRM chromatograms for 84-B10 and Ginkgolide J (IS) from (**A**) blank rat plasma; (**B**) blank rat plasma spiked with 84-B10 (1 ng/mL) and Ginkgolide J (2 μg/mL); (**C**) plasma samples 1 h after injection of 84-B10.

Multiple reaction monitor (MRM) was selected for accurate quantification. The response was higher in negative mode due to the presence of carboxylic acid. The product ions were the acquired, and the results are shown in Figure 1. 472.0/282.0 and 423.0/349.2 were selected as the quantitative ion pairs for the analyte and internal standard, respectively. Figure 2 shows the representative MRM chromatograms of 84-B10 and the internal standard. The retention time was 1.41 min and 1.24 min, respectively. No significant interferences were observed, indicating a good specificity of the method. The linear ranges for 84-B10 in different biological matrices are shown in Table 1, and the correlation coefficients (r) were all greater than 0.99, indicating a good linearity. The intra-day and inter-day relative standard deviation (RSD) and relative error (RE) were less than 15% (Table 2), suggesting adequate precision and accuracy. Table 3 shows the matrix effects and recoveries of 84-B10 and IS in rat plasma. For the matrix effect, the RSD values of 84-B10 and IS were 3.68% and 12.2%. The recovery of 84-B10 ranged from 92% to 95%, with RSD less than 6.02%. Recovery of IS was 85%, with RSD of 2.53% (Table 3). The matrix had no obvious effect on the accuracy of the method and the extraction recovery was acceptable. Table 4 shows the stability evaluation of 84-B10 under different storage conditions, such as room temperature for 2 h, autosampler for 24 h and three freeze–thaw cycles. Specifically, both RSD (%) and RE (%) were less than 11%, demonstrating a certifiable stability.

### 2.2. Pharmacokinetic and Tissue Distribution of 84-B10 in Rats

**Figure 3:**
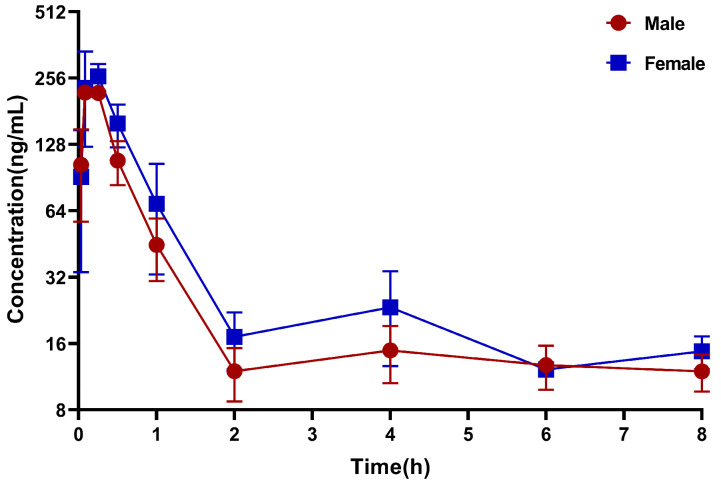
Mean plasma concentration–time curves after a single intraperitoneal injection of 0.36 mg/kg 84-B10 (mean ± SD, n = 3).

**Figure 4:**
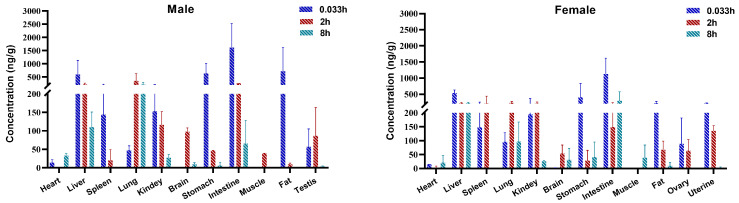
Tissue distribution after a single intraperitoneal injection of 0.36 mg/kg 84-B10 (mean ± SD, n = 3).

The validated UPLC-MS/MS method was successfully applied to the pharmacokinetic study and tissue distribution in rats after an intraperitoneal injection of 84-B10 (0.36 mg/kg). Figure 3 displays the mean plasma concentration–time curves and Table 5 summarizes the corresponding pharmacokinetic parameters. According to our study, there was no significant gender difference in the absorption of 84-B10. After intraperitoneal injection, 84-B10 reached its peak concentration quickly, with a t_max_ of 0.17 h and a C_max_ of 257 ng/mL. The elimination half-life (t_1/2_) was 0.44 h and the clearance rate was 1329 mL/h/kg, indicating a rapid elimination of B10 in plasma. Two hours later, the plasma concentration tended to flatten out, with a concentration of approximately 10 ng/mL. AUC_0–t_ and AUC_0–∞_ were 269 and 278 h*ng/mL, respectively. After an intraperitoneal administration, 84-B10 could be detected in different tissues, indicating a wide distribution (Figure 4). The compound rapidly distributed and reached maximum concentration at 0.033 h for most tissues. 84-B10 was most concentrated in the intestine, stomach, liver, kidney and lung, which was consistent with high expression of LONP1 in those tissues. In most tissues, 84-B10 was eliminated at 8 h.

### 2.3. PBPK Modeling of 84-B10

**Figure 5:**
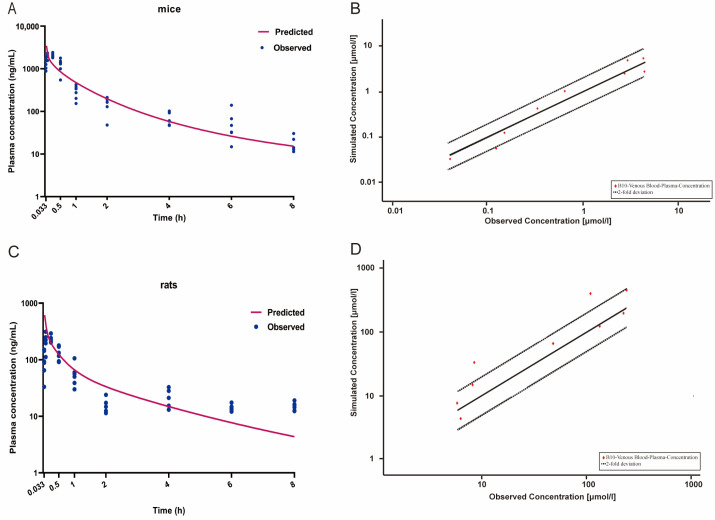
Simulated (lines) and observed (filled dots) plasma concentrations of 84-B10 after an intraperitoneal injection in (**A**) mice (5 mg/kg) and (**C**) rats (0.36 mg/kg). Relationship of mean observed and predicted plasma concentration at each time point in (**B**) mice and (**D**) rats, where solid and dashed lines indicate unity and two-fold errors between predicted and observed data, respectively.

The model was constructed using PK-Sim (version 11.1, Bayer Technology Services, Leverkusen, Germany). The lung, heart, spleen, gastrointestinal tract, liver, kidney, brain, adipose, muscle, skin, arterial and venous blood, as well as other parts of the body, were used as the essential structures. Input physicochemical parameters are listed in Table 6. Molecular weight and pKa of the compound were obtained from Chemdraw (version 19.0, PerkinElmer Informatics, Inc., Waltham, MA, USA). Log P, permeability, aqueous solubility clearance, and fraction unbound were acquired from ADMET lab 2.0 (https://admetmesh.scbdd.com/, accessed on 31 July 2023). Other parameters such as blood/plasma concentration ratio, partition coefficients, cellular permeabilities, various tissue volumes and blood flow were optimized via PK-Sim. The observed plasma concentrations of 84-B10 in mice were acquired from our previous publication [14] and the values were converted into ng/mL units. The predicted concentrations were simulated using the developed PBPK model (Figure 5A) and the predicted curve commendably reflected the overall trend of the measured pharmacokinetic curve. Goodness-of-fit plots also indicated a successful prediction (Figure 5B). Similarly, the model was capable of predicting the plasma concentration of 84-B10 in rats and was validated with experimental data (Figure 5C,D). The difference between predicted and measured concentrations at most time points was within twice the error. Corresponding PK parameters of observed data were calculated using the non-compartmental model of WinNonlin 7.0, while those of predicted data were automatically obtained using PK-Sim (Table 7). The prediction accuracy was evaluated using fold error, which is equal to the ratio of predicted data to observed data. Typically, when the fold error is between 0.5 to 2, the prediction is considered accurate. 

### 2.4. Extrapolation of PBPK Model to Humans

**Figure 6:**
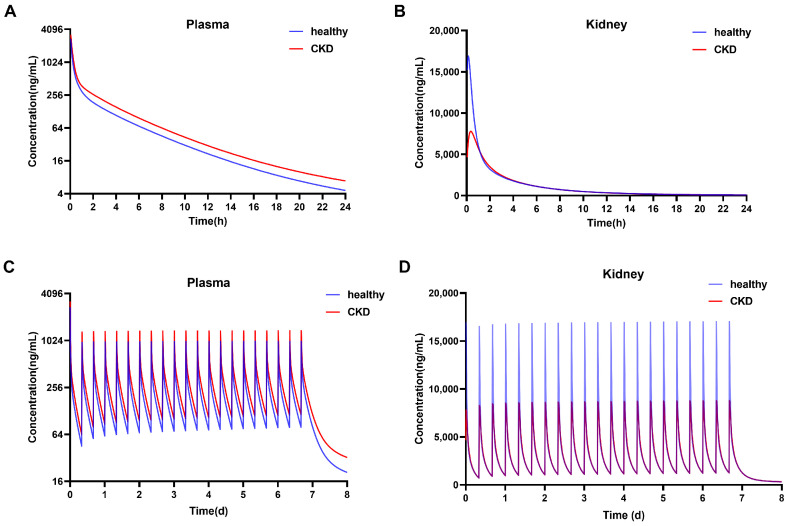
The simulated concentrations after (**A**,**B**) a single and (**C**,**D**) multiple dose of 84-B10 (0.41 mg/kg) in plasma and kidney of healthy individuals and CKD patients.

We replaced the physiological parameters of animals with those of humans and incorporated them into the PBPK model. Healthy European male subjects were selected, with an average age of 30 years, a body weight of 73 kg and a BMI of 23.57 kg·m^−2^. The drug concentration in healthy individuals and CKD patients after a single intravenous injection of 0.41 mg/kg of 84-B10 was simulated (Figure 6A,B). As renal blood flow and glomerular filtration rate decreased significantly in severe CKD patients, the elimination of 84-B10 was slower than that of healthy individuals. In plasma, T_1/2_ was 8.31 versus 7.88 h, C_max_ was 3250 versus 2735 ng/mL, and AUC_0–t_ was 306 versus 227 μmol*min/L. C_max_ in the kidney of CKD patients was 7787 ng/mL, which was much lower than that of healthy volunteers (16,888 ng/mL). When administrated every 8 h, it reached a steady state the next day. C_max,ss_ in the plasma of CKD patients was 1360 ng/mL, and 997 ng/mL in healthy volunteers (Figure 6C). C_max,ss_ in the kidney of CKD patients was 8773 ng/mL, while it was 17,010 ng/mL in healthy individuals (Figure 6D). 

## 3. Discussion

LonP1 is a protease that resides in the matrix and participates in oxidative stress and mitochondrial autophagy regulation, which is important for maintaining mitochondrial and cellular homeostasis [27]. LonP1 can protect myocardial cells from ischemia/reperfusion injury [28], and help lung fibroblasts maintain normal cell viability [29]. Recently, its role in CKD pathogenesis has been elucidated. LonP1 was identified as an endogenous mitochondrial regulator in renal tubular cells under CKD conditions and its deletion could impair mitochondrial homeostasis and aggravated renal fibrosis through the substrate accumulation. 84-B10 was identified as a new LonP1 activator and has been proved to attenuate renal fibrosis and mitochondrial dysfunction [14].

In this study, we aimed to investigate the pharmacokinetics of 84-B10 based on previous research. A rapid and accurate quantification method by UHPLC-MS/MS was validated first. A full scan of 84-B10 was performed in both positive and negative modes, and the higher response in negative mode was due to the presence of carboxylic acid. Ginkgolide J was selected as the internal standard due to its similarity in molecular weight and chromatographic retention behavior to 84-B10. There are three benzene rings in the structure of 84-B10, with which the bonding phase of the phenyl column can form pi–pi conjugations. Therefore, the chromatographic retention of the aromatic compound was improved and unique selectivity could be provided. Subsequently, the method was validated. According to *Bioanalytical Method Validation Guidance for Industry* issued by the FDA in 2018, the selectivity, sensitivity, linearity, precision, accuracy, recovery, matrix effect and stability met the requirement. The pharmacokinetic characteristics of 84-B10 were studied using the validated method and actual plasma concentrations were obtained for the following PBPK model verification. 

Furthermore, the PK behavior was stimulated by the PBPK model using in silico data. The simulation quality depended on the input parameters, and the sensitivity parameter analysis of the model showed that fraction unbound in plasma, lipophilicity, and permeability had significant impact on AUC and C_max_. After optimizing these parameters, the PBPK model was successfully constructed and validated with experimental data from mice and rats. The model was then applied to predict the plasma concentration in clinical practice. Previous studies have indicated that 5 mg/kg of 84-B10 has a significant therapeutic effect on cisplatin-induced acute kidney injury in mice. In addition, 84-B10 dose-dependently alleviated renal fibrosis induced by unilateral ureteral obstruction (UUO) or unilateral ischemia reperfusion injury (UIRI) in mice at a dose of 0.5–7.5 mg/kg [13,14]. On this basis, the PBPK model was used to predict PK parameters in healthy volunteers and CKD patients after administration of 84-B10 at 0.41 mg/kg. It was estimated that the plasma C_max_ in CKD patients was 3250 ng/mL after a single dose of 84-B10 (0.41 mg/kg) and C_max,ss_ was 1360 ng/mL after multiple doses. Kidney C_max_ and C_max,ss_ were 7787 ng/mL and 8773 ng/mL, which were much higher than those in plasma. Previous research has shown that the binding affinity of 84-B10 is 312.5 nM (147 ng/mL) [14], and our study indicated that the drug concentration in the kidneys remains at 150 ng/mL until 17 h after administration, suggesting a sufficient effect in CKD patients. Certainly, our research on human PK is just a model-based deduction, and in the future, actual clinical samples will be needed to verify the accuracy of the model. Due to the fact that the concentration of 84-B10 in the kidney may be much higher than that in plasma, more toxicological tests are needed to ensure its safety in clinical application. 

## 4. Materials and Methods

### 4.1. Chemicals and Reagents

84-B10 (purity > 99%) was synthesized by Shanghai Medicilon Inc. (Shanghai, China). Ginkgolide J (purity > 99%) was purchased from Chengdu Pufei De Biotech Co., Ltd. (Chengdu, China). HPLC-grade methanol was purchased from Sigma-Aldrich (Shanghai, China). Ammonium formate was bought from Fisher Scientific (Pittsburgh, PA, USA). Dimethyl sulfoxide (AR) was obtained from Sinopharm Chemical Reagent Co., Ltd. (Shanghai, China). Ultrapure water was prepared by the Milli-Q Ultrapure water purification system (Millipore, Bedford, MA, USA).

### 4.2. Laboratory Animals

Sprague Dawley rats (10 weeks old, 300 g) and C57BL/6J mice (8 weeks old, 20 g) were purchased from Jinan Pengyue Laboratory Animal Technology Co., Ltd. (Jinan, China) and were kept in the animal feeding room with a temperature of 22 ± 2 °C and a humidity of 55 ± 5% in a 12 h normal light/dark cycle. The study was approved by the ethics committee of Yantai University to guarantee animal welfare.

### 4.3. UHPLC-MS/MS Method Validation for the Quantification of 84-B10 

84-B10 was determined and quantified by ExionLC system coupled with SCIEX 4500 triple quadrupole mass spectrometers (SCIEX, Framingham, MA, USA). A phenyl HPLC column (50 × 2.1 mm, 3 μm) (YMC, Kyoto, Japan) was used for separation. Methanol was selected as the organic phase as it could better reflect the π-π selectivity between benzene and the chromatographic column. An amount of 5 mM ammonium formate was added into water to adjust the dissociation of 84-B10 and to improve the chromatographic peak. The flow rate was 0.3 mL/min with gradient elution (Table 8). For mass detection, the optimized parameters are listed in Table 9. 

The method was validated according to the *Bioanalytical Method Validation Guidance for Industry* issued by Food and Drug Administration of USA in 2018. In short, blank plasma from six different rats was collected and spiked with or without LLOQ working solutions to evaluate the sensitivity. The linear least square method was used to fit the standard curve, and the weighting coefficient was 1/x^2^. The precision and accuracy were evaluated by analyzing three different concentrations of quality control samples in three consecutive days. Blank plasma was first precipitated with methanol and then spiked with QC working solutions into supernatant. The peak area was compared with QC samples to estimate the extraction recovery. Alternatively, blank plasma was replaced with ultrapure water and mixed with QC working solutions. The matrix effect was then evaluated. For stability evaluation, different concentrations of QC samples were stored at room temperature for 2 h, frozen–thawed for three cycles, or stored in an auto-sampler (extracted samples, 4 °C) for 24 h and then analyzed. 

### 4.4. Pharmacokinetic and Tissue Distribution 

After one week of adaptive feeding, pharmacokinetic experiments were conducted. 84-B10 (0.36 mg/kg) solution was injected and blood was collected into tubes containing heparin sodium at 0.033, 0.083, 0.25, 0.5, 1, 2, 4, 6 and 8 h after administration. Blood was centrifuged at 8000 rpm for 5 min to obtain the plasma. The tissue was accurately weighed and ground with pure water (*w*:*v*, 1:5) to obtain homogenate. An amount of 50 µL of biological matrix was precipitated with 200 μL of methanol containing IS (Ginkgolide J, 2 μg/mL). After vortexing for 5 min, the samples were centrifuged at 12,000 rpm for 5 min and the supernatant was taken for injection. 

### 4.5. PBPK Modeling

The model was constructed using PK-Sim (http://open-systems-pharmacology.org, accessed 20 August 2023) and lung, heart, spleen, gastrointestinal tract, liver, kidney, brain, adipose, muscle, skin, arterial and venous blood, and rest of body, as the essential structure. Pharmacokinetic profiles in mice and rats were predicted and the parameters were estimated using non-compartmental analysis. The fold error was calculated to evaluate the goodness of fit [30]. After optimization, the model was extrapolated to predict pharmacokinetic profiles in humans. For healthy individuals, the physiological parameters were defined as the default value of healthy European male subjects. For server CKD patients, the physiological parameters were set according to the literature [31,32,33]. The physicochemical parameters and biochemical properties of the compound were acquired via ADMETlab 2.0 (https://admetmesh.scbdd.com/, accessed 31 July 2023) or ChemDraw 19.0 software.

## 5. Conclusions

Previous studies have demonstrated that 84-B10, a novel 3-phenylglutaric acid derivative, has the potential to treat AKI and CKD. However, there is little information about its pharmacokinetics. The UHPLC-MS/MS method for the quantification of 84-B10 was validated first in this study and then was applied to explore the pharmacokinetic profile in rats to obtain adequate parameters. A PBPK model was established via in silico data and then was validated using the experimental data from mice and rats. The optimized model was used to predict PK in healthy volunteers and CKD patients after a single or multiple dose. The results indicated an adequate efficacy of 84-B10. In summary, our research provides supplemental pharmacokinetic study and PBPK application for 84-B10, and may serve in clinical dosage setting in the future.

## Figures and Tables

**Table 1:** The calibration curve, correlation coefficient (r), linear range and lower limit of quantification (LLOQ) of 84-B10 in rat biological samples.

Biological Matrix	Calibration Curve	r	Linear Range (ng/mL)	LLOQ (ng/mL)
Plasma	y = 45.259x − 0.9096	0.9997	1–1000	1
Heart	y = 80.738x + 0.4783	0.9981	1–1000	1
Liver	y = 80.562x − 8.0523	0.9908	1–1000	1
Spleen	y = 34.642x − 4.2273	0.9973	10–1000	10
Lung	y = 84.462x + 8.2483	0.9907	1–1000	1
Kidney	y = 70.598x + 9.66	0.9929	1–1000	1
Brain	y = 30.828x + 1.3522	0.9999	10–1000	10
Stomach	y = 32.986x − 12.094	0.9997	10–1000	10
Intestine	y = 79.144x + 0.6974	0.9975	1–1000	1
Muscle	y = 35.05x − 17.483	0.9993	10–1000	10
Fat	y = 40.822x − 0.2159	0.9998	10–1000	10
Testis	y = 41.882x + 7.4713	0.9997	10–1000	10
Ovary	y = 44.511x − 4.902	0.9969	10–1000	10
Uterine	y = 38.183x − 9.2433	0.9996	10–1000	10

**Table 2:** Intra-day and inter-day precision and accuracy of 84-B10 in rat plasma.

	Normalized Concentration (ng/mL)	1	3	300	750
Day 1 (n = 6)	Measured (Mean ± SD)	0.99 ± 0.10	3.29 ± 0.09	301 ± 7	683 ± 30
	RSD (%)	10.6	2.87	2.38	4.30
	RE (%)	−1.12	9.67	0.22	−8.93
Day 2 (n = 6)	Measured (Mean ± SD)	1.10 ± 0.13	3.31 ± 0.08	286 ± 39	703 ± 19
	RSD (%)	11.3	2.35	13.5	2.71
	RE (%)	10.3	10.2	−4.83	−6.24
Day 3 (n = 6)	Measured (Mean ± SD)	0.91 ± 0.08	3.34 ± 0.10	319 ± 20	733 ± 18
	RSD (%)	8.70	2.91	6.22	2.34
	RE (%)	−9.02	11.2	6.44	−2.31
Inter-day (n = 18)	Measured (Mean ± SD)	1.01 ± 0.13	3.31 ± 0.09	304 ± 25	706 ± 30
	RSD (%)	13.0	2.62	8.30	4.22
	RE (%)	0.83	10.3	1.29	−5.83

**Table 3:** Matrix effect and extraction recovery for 84-B10 and IS in rat plasma (n = 6).

Normalized Concentration (ng/mL)	Matrix Effect (%) (Mean ± SD)	RSD (%)	Recovery (%) (Mean ± SD)	RSD (%)
3	135 ± 7		92 ± 6	
300	129 ± 3	3.68	93 ± 4	6.03
750	130 ± 5		95 ± 8	
2000 (IS)	92.0 ± 2.3	12.2	85 ± 1	2.53

**Table 4:** Stability of 84-B10 in rat plasma under different conditions (n = 6).

Storage Conditions	Spiked Concentration (ng/mL)	Measured Concentrations (ng/mL)	RSD (%)	RE (%)
Room temperature for 2 h	3	2.96 ± 0.10	3.29	−1.50
	300	278 ± 3	1.20	−7.44
	750	670 ± 15	2.23	−10.7
Autosampler stability (4 °C for 24 h)	3	3.15 ± 0.11	3.34	5.00
	300	299 ± 10	3.45	−0.33
	750	710 ± 27	3.75	−5.38
Three freeze–thaw cycles	3	2.87 ± 0.29	9.99	−4.22
	300	287 ± 20	6.97	−4.28
	750	684 ± 18	2.59	−8.87

**Table 5:** Main pharmacokinetic parameters in rats after a single intraperitoneal injection of 0.36 mg/kg 84-B10 (mean ± SD).

	T_1/2_ (h)	CL (mL/h/kg)	T_max_ (h)	C_max_ (ng/mL)	AUC_0–t_ (h*ng/mL)	AUC_0–∞_ (h*ng/mL)
Male	0.45 ± 0.02	1149 ± 119	0.19 ± 0.10	284 ± 48.2	305 ± 30.2	315 ± 31.0
Female	0.43 ± 0.04	1509 ± 177	0.14 ± 0.10	231 ± 2.08	233 ± 27.0	241 ± 29.0
Total	0.44 ± 0.03	1329 ± 239	0.17 ± 0.09	257 ± 42.4	269 ± 47.3	278 ± 48.9

**Table 6:** Input parameters used in the PBPK model for 84-B10.

Parameter	Predicted Value	Source and Reference
Molecular weight (g/mol)	473.15	Chemdraw
pKa (acid)	4.22	Chemdraw
Log P	4.82	ADMET lab 2.0
Permeability (10^−6^ cm/s)	9.30	ADMET lab 2.0
Aqueous solubility at pH 7 (μg/mL)	1.75	ADMET lab 2.0
Fu (Fraction unbound)	0.61%	ADMET lab 2.0
CLH (mL/min/kg)	4.26	ADMET lab 2.0
CLR (mL/min/kg)	4.26	ADMET lab 2.0
Blood/Plasma concentration ratio	1.50	PK-Sim
Partition coefficients		PK-Sim standard
Cellular permeabilities		PK-Sim standard

**Table 7:** Observed and predicted pharmacokinetic parameters of 84-B10 in mice and rats.

	Mice			Rats		
	Observed	Predicted	Fold Error	Observed	Predicted	Fold Error
C_max_ (ng/mL)	2038	3464	1.70	257	615	2.39
AUC_0→t_ (h*ng/mL)	1327	1599	1.20	269	260	0.97
V_d_ (L/kg)	11.20	12.96	1.16	4.13	4.50	1.09
Cl (mL/min/kg)	56.8	47.5	0.84	22.2	19.7	0.89

**Table 8:** Gradient program for the quantification of 84-B10 and IS.

Time (min)	Water (5 mM Ammonium Formate, %)	Methanol (%)
0.1	80	20
0.3	5	95
1.8	5	95
1.9	80	20
3.0	stop	stop

**Table 9:** Mass parameters for the quantification of 84-B10 and IS.

Compounds	Precursor Ion	Product Ion	CE (eV)	DP (eV)
84-B10	472.0	282.0	−30	−120
IS	423.0	349.0	−40	−130

## Data Availability

Data are available from the authors.

## References

[1] Naber T., Purohit S. (2021). Chronic Kidney Disease: Role of Diet for a Reduction in the Severity of the Disease. Nutrients.

[2] Levey A.S., James M.T. (2017). Acute Kidney Injury. Ann. Intern. Med..

[3] Charles C., Ferris A.H. (2020). Chronic Kidney Disease. Prim. Care.

[4] Vallianou N.G., Mitesh S., Gkogkou A., Geladari E. (2019). Chronic Kidney Disease and Cardiovascular Disease: Is there Any Relationship?. Curr. Cardiol. Rev..

[5] GBD Chronic Kidney Disease Collaboration (2020). Global, Regional, and National Burden of Chronic Kidney Disease, 1990–2017: A Systematic Analysis for the Global Burden of Disease Study 2017. Lancet.

[6] Evans M., Lewis R.D., Morgan A.R., Whyte M.B., Hanif W., Bain S.C., Davies S., Dashora U., Yousef Z., Patel D.C. (2022). A Narrative Review of Chronic Kidney Disease in Clinical Practice: Current Challenges and Future Perspectives. Adv. Ther..

[7] Chen T.K., Knicely D.H., Grams M.E. (2019). Chronic Kidney Disease Diagnosis and Management: A Review. JAMA.

[8] Mende C.W. (2022). Chronic Kidney Disease and SGLT2 Inhibitors: A Review of the Evolving Treatment Landscape. Adv. Ther..

[9] Zhu Z., Hu J., Chen Z., Feng J., Yang X., Liang W., Ding G. (2022). Transition of Acute Kidney Injury to Chronic Kidney Disease: Role of Metabolic Reprogramming. Metabolism.

[10] Chawla L.S., Eggers P.W., Star R.A., Kimmel P.L. (2014). Acute Kidney Injury and Chronic Kidney Disease as Interconnected Syndromes. N. Engl. J. Med..

[11] Ferenbach D.A., Bonventre J.V. (2016). Acute Kidney Injury and Chronic Kidney Disease: From the Laboratory to the Clinic. Nephrol. Ther..

[12] Zhang X., Agborbesong E., Li X. (2021). The Role of Mitochondria in Acute Kidney Injury and Chronic Kidney Disease and Its Therapeutic Potential. Int. J. Mol. Sci..

[13] Fan J., Xu X., Li Y., Zhang L., Miao M., Niu Y., Zhang Y., Zhang A., Jia Z., Wu M. (2023). A Novel 3-phenylglutaric Acid Derivative (84-B10) Alleviates Cisplatin-Induced Acute Kidney Injury by Inhibiting Mitochondrial Oxidative Stress-Mediated Ferroptosis. Free Radic. Biol. Med..

[14] Bai M., Wu M., Jiang M., He J., Deng X., Xu S., Fan J., Miao M., Wang T., Li Y. (2023). LONP1 Targets HMGCS2 to Protect Mitochondrial Function and Attenuate Chronic Kidney Disease. EMBO Mol. Med..

[15] Knoll K.E., van der Walt M.M., Loots D.T. (2022). In Silico Drug Discovery Strategies Identified ADMET Properties of Decoquinate RMB041 and Its Potential Drug Targets Against Mycobacterium Tuberculosis. Microbiol. Spectr..

[16] El Fadili M., Er-Rajy M., Kara M., Assouguem A., Belhassan A., Alotaibi A., Mrabti N.N., Fidan H., Ullah R., Ercisli S. (2022). QSAR, ADMET In Silico Pharmacokinetics, Molecular Docking and Molecular Dynamics Studies of Novel Bicyclo (Aryl Methyl) Benzamides as Potent GlyT1 Inhibitors for the Treatment of Schizophrenia. Pharmaceuticals.

[17] Zhuang X., Lu C. (2016). PBPK Modeling and Simulation in Drug Research and Development. Acta Pharm. Sin. B.

[18] Tan Y.M., Chan M., Chukwudebe A., Domoradzki J., Fisher J., Hack C.E., Hinderliter P., Hirasawa K., Leonard J., Lumen A. (2020). PBPK Model Reporting Template for Chemical Risk Assessment Applications. Regul. Toxicol. Pharmacol..

[19] Sager J.E., Yu J.J., Ragueneau-Majlessi I., Isoherranen N. (2015). Physiologically Based Pharmacokinetic (PBPK) Modeling and Simulation Approaches: A Systematic Review of Published Models, Applications, and Model Verification. Drug Metab. Dispos..

[20] Li X., Jusko W.J. (2022). Assessing Liver-to-Plasma Partition Coefficients and in Silico Calculation Methods: When Does the Hepatic Model Matter in PBPK?. Drug Metab. Dispos..

[21] Yuan D., He H., Wu Y., Fan J., Cao Y.G. (2019). Physiologically Based Pharmacokinetic Modeling of Nanoparticles. J. Pharm. Sci..

[22] Niederalt C., Kuepfer L., Solodenko J., Eissing T., Siegmund H., Block M., Willmann S., Lippert J. (2018). A Generic Whole Body Physiologically Based Pharmacokinetic Model for Therapeutic Proteins in PK-Sim. J. Pharmacokinet. Pharmacodyn..

[23] Deepika D., Kumar V. (2023). The Role of “Physiologically Based Pharmacokinetic Model (PBPK)” New Approach Methodology (NAM) in Pharmaceuticals and Environmental Chemical Risk Assessment. Int. J. Env. Res. Public. Health.

[24] Chen J.R., You X., Wu W.H., Guo G.M., Lin R.F., Ke M., Huang P.F., Lin C.H. (2023). Application of PBPK Modeling in Predicting Maternal and Fetal Pharmacokinetics of Levetiracetam During Pregnancy. Eur. J. Pharm. Sci..

[25] Bi Y.W., Deng J.X., Murry D.J., An G.H. (2016). A Whole-Body Physiologically Based Pharmacokinetic Model of Gefitinib in Mice and Scale-Up to Humans. AAPS J..

[26] Wachsmuth L., Mensen A., Barca C., Wiart M., Tristão-Pereira C., Busato A., Waiczies S., Himmelreich U., Millward J.M., Reimann H.M. (2021). Contribution of Preclinical MRI to Responsible Animal Research: Living Up to the 3R Principle. MAGMA.

[27] Bahat A., Perlberg S., Melamed-Book N., Isaac S., Eden A., Lauria I., Langer T., Orly J. (2015). Transcriptional Activation of LON Gene by a New Form of Mitochondrial Stress: A Role for the Nuclear Respiratory Factor 2 in StAR Overload Response (SOR). Mol. Cell Endocrinol..

[28] Venkatesh S., Li M., Saito T., Tong M.M., Rashed E., Mareedu S., Zhai P.Y., Bárcena C., López-Otín C., Yehia G. (2019). Mitochondrial LonP1 Protects Cardiomyocytes from Ischemia/Reperfusion Injury in Vivo. J. Mol. Cell Cardiol..

[29] Ngo J.K., Pomatto L.C., Bota D.A., Koop A.L., Davies K.J. (2011). Impairment of Ion-Induced Protection Against the Accumulation of Oxidized Proteins in Senescent wi-38 Fibroblasts. J. Gerontol. A Biol. Sci. Med. Sci..

[30] Yamazaki S.J., Skaptason J., Romero D., Vekich S., Jones H.M., Tan W.W., Wilner K.D., Koudriakova T. (2011). Prediction of Oral Pharmacokinetics of cMet Kinase Inhibitors in Humans: Physiologically Based Pharmacokinetic Model Versus Traditional one-Compartment Model. Drug Metab. Dispos..

[31] Willmann S., Coboeken k., Kapsa S., Thelen K., Mundhenke M., Fischer K., Hügl B., Mück W. (2021). Applications of Physiologically Based Pharmacokinetic Modeling of Rivaroxaban-Renal and Hepatic Impairment and Drug-Drug Interaction Potential. J. Clin. Pharmacol..

[32] Hafsa H., Zamir A., Rasool M.F., Imran I., Saeed H., Ahmad T., Alsanea S., Alshamrani A.A., Alruwaili A.H., Alqahtani F. (2022). Development and Evaluation of a Physiologically Based Pharmacokinetic Model of Labetalol in Healthy and Diseased Populations. Pharmaceutics.

[33] Heimbach T., Chen Y., Chen J., Dixit V., Parrott N., Peters S.H., Poggesi I., Sharma P., Snoeys J., Shebley M. (2021). Physiologically-Based Pharmacokinetic Modeling in Renal and Hepatic Impairment Populations: A Pharmaceutical Industry Perspective. Clin. Pharmacol. Ther..

